# Dichloridobis(pyridine-2-thiol­ato-κ^2^ *N*,*S*)tin(IV): a new polymorph

**DOI:** 10.1107/S1600536812024026

**Published:** 2012-06-13

**Authors:** Sheyda R. Ismaylova, Zhanna V. Matsulevich, Galina N. Borisova, Alexander V. Borisov, Victor N. Khrustalev

**Affiliations:** a Baku State University, Z. Khalilov St 23, Baku AZ-1148, Azerbaijan; b R.E. Alekseev Nizhny Novgorod State Technical University, 24 Minin St, Nizhny Novgorod 603950, Russian Federation; c X-Ray Structural Centre, A.N. Nesmeyanov Institute of Organoelement Compounds, Russian Academy of Sciences, 28 Vavilov St, B-334, Moscow 119991, Russian Federation

## Abstract

The title compound, [SnCl_2_(C_5_H_4_NS)_2_], is the product of reaction of 2,2′-dipyridyl disulfide with tin tetra­chloride. The Sn^IV^ atom adopts a distorted octa­hedral geometry, with the two bidentate pyridine-2-thiol­ate ligands forming two planar four-membered chelate rings. The two Sn—Cl, two Sn—N and two Sn—S bonds are in *cis*, *cis* and *trans* configurations, respectively. The crystal grown from acetonitrile represents a new monoclinic polymorph in space group *C*2/*c* with the mol­ecule having twofold rotational symmetry, the Sn^IV^ atom lying on the twofold axis. The mol­ecular structure of the monoclinic polymorph is very close to that of the triclinic polymorph studied previously in space group *P*-1, the mol­ecule occupying a general position [Masaki & Matsunami (1976 ▶). *Bull. Chem. Soc. Jpn*, **49**, 3274–3279; Masaki *et al.* (1978 ▶). *Bull. Chem. Soc. Jpn*, **51**, 3298–3301]. Apparently, the formation of the two polymorphs is determined by the different systems of inter­molecular inter­actions. In the crystal of the monoclinic polymorph, mol­ecules are bound into ribbons along the *c* axis by C—H⋯Cl hydrogen bonds, whereas in the crystal of the triclinic polymorph, mol­ecules form chains along the *a* axis by attractive S⋯S inter­actions. The crystal studied was a pseudo-merohedral twin; the refined BASF value is 0.221 (1).

## Related literature

For metal complexes with 2,2′-dipyridyl dichalcogenides, see: Kadooka *et al.* (1976*a* ▶,*b* ▶); Cheng *et al.* (1996 ▶); Kienitz *et al.* (1996 ▶); Bell *et al.* (2000 ▶); Kita *et al.* (2001 ▶); Kedarnath *et al.* (2009 ▶). For the triclinic polymorph, see: Masaki & Matsunami (1976 ▶); Masaki *et al.* (1978 ▶).
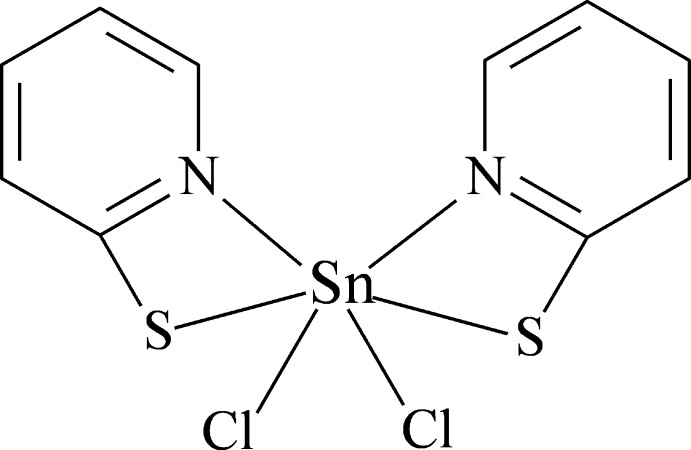


## Experimental

### 

#### Crystal data

[SnCl_2_(C_5_H_4_NS)_2_]
*M* *_r_* = 409.93
Monoclinic, 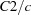

*a* = 6.3240 (7) Å
*b* = 12.9391 (14) Å
*c* = 16.4240 (18) Å
β = 100.922 (2)°
*V* = 1319.6 (3) Å^3^
*Z* = 4
Mo *K*α radiation
μ = 2.63 mm^−1^
*T* = 100 K
0.16 × 0.14 × 0.10 mm

#### Data collection

Bruker SMART 1K CCD diffractometer
Absorption correction: multi-scan (*SADABS*; Sheldrick, 1998 ▶) *T*_min_ = 0.678, *T*_max_ = 0.779
6681 measured reflections
1584 independent reflections
1562 reflections with *I* > 2σ(*I*)
*R*_int_ = 0.024

#### Refinement

*R*[*F* ^2^ > 2σ(*F* ^2^)] = 0.020
*wR*(*F* ^2^) = 0.050
*S* = 1.00
1584 reflections
79 parameters
H-atom parameters constrained
Δρ_max_ = 0.81 e Å^−3^
Δρ_min_ = −0.53 e Å^−3^

### 

Data collection: *SMART* (Bruker, 1998 ▶); cell refinement: *SAINT* (Bruker, 1998 ▶); data reduction: *SAINT*; program(s) used to solve structure: *SHELXTL* (Sheldrick, 2008 ▶); program(s) used to refine structure: *SHELXTL*; molecular graphics: *SHELXTL*; software used to prepare material for publication: *SHELXTL*.

## Supplementary Material

Crystal structure: contains datablock(s) global, I. DOI: 10.1107/S1600536812024026/rk2356sup1.cif

Structure factors: contains datablock(s) I. DOI: 10.1107/S1600536812024026/rk2356Isup2.hkl

Additional supplementary materials:  crystallographic information; 3D view; checkCIF report

## Figures and Tables

**Table 1:** Hydrogen-bond geometry (Å, °)

*D*—H⋯*A*	*D*—H	H⋯*A*	*D*⋯*A*	*D*—H⋯*A*
C3—H3⋯Cl1^i^	0.95	2.80	3.673 (3)	154

Symmetry code: (i) 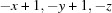.

**Fig. 1.**
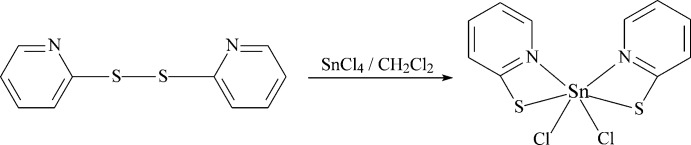
Reaction of 2,2'-dipyridyl disulfide with tin tetrachloride.

**Fig. 2.**
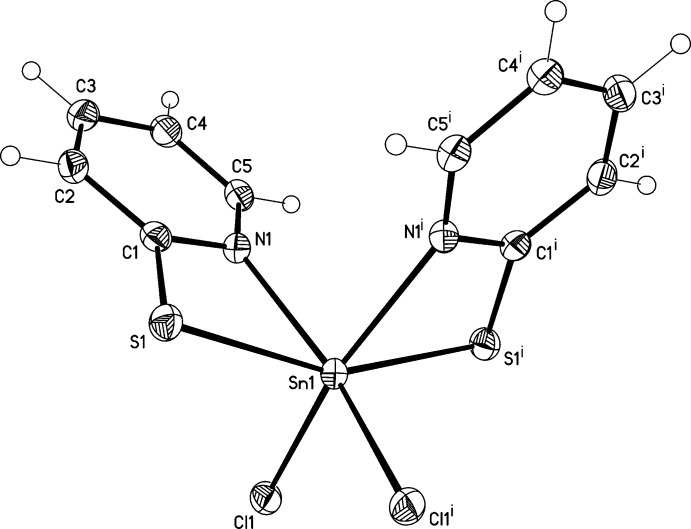
Molecular structure of I with the atom numbering scheme. Displacement ellipsoids are shown at the 50% probability level. H atoms are presented as a small spheres of arbitrary radius. Symmetry code: (i) -x+1, y, -z+1/2.

**Fig. 3.**
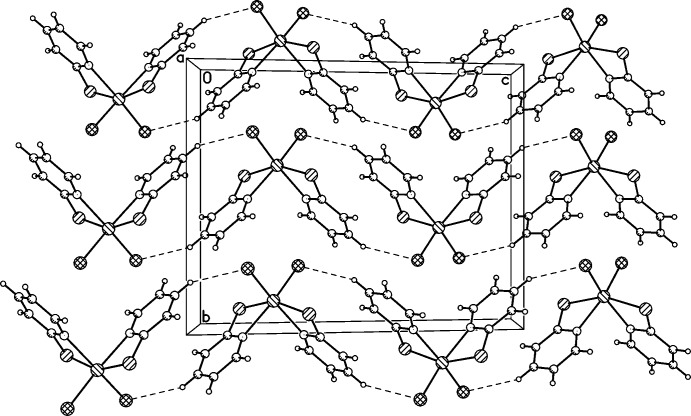
The H-bonded ribbons along the c axis in the monoclinic polymorph of I.

**Fig. 4.**
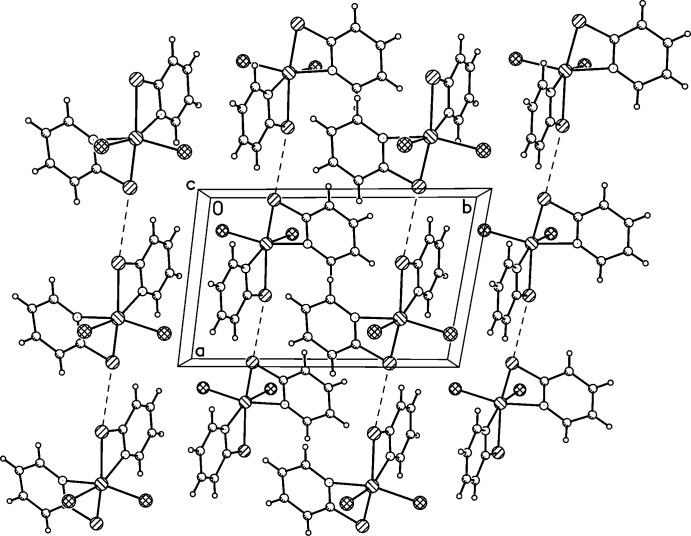
The S···S bonded chains along the a axis in the triclinic polymorph of I. Dashed lines indicate the intermolecular non-valent interactions.

## supplementary crystallographic information

### Comment

The coordination chemistry of 2,2'-dipyridyl dichalcogenides to metal ions is a topic of current research interest owing to the application of these complexes as potential precursors for the generation of semiconducting materials (Kadooka *et al.*, 1976*a*, 1976*b*; Cheng *et al.*, 1996; Kienitz *et al.*, 1996; Bell *et al.*, 2000; Kita *et al.*, 2001; Kedarnath *et al.*, 2009).

This article describes the new monoclinic polymorph of dichlorobis(2-pyridinethiolato)tin(IV), C_10_H_8_Cl_2_N_2_S_2_Sn (I), which was obtained by the reaction of 2,2'-dipyridyl disulfide with tin tetrachloride (Fig. 1). The synthesis of the title compound by the reaction of 2,2'-dipyridyl disulfide with tin dichloride and its triclinic polymorph were reported previously (Masaki & Matsunami, 1976; Masaki *et al.*, 1978).

The molecule of I possesses overall intrinsic *C*_2_ symmetry. In contrast to the triclinic polymorph (the space group *P*1, the molecule occupies a common position), this symmetry is realised in the crystal of the monoclinic polymorph (the space group *C*2/*c*, the molecule occupies a special position on the twofold axis). The tin atom adopts a distorted octahedral geometry, with the two bidentate 2-pyridinethiolato ligands forming two planar four-membered chelate rings (Fig. 2). The two Sn–Cl, two Sn–N and two Sn–S bonds are in *cis*-, *cis*- and *trans*-configurations, respectively. Generally, the molecular structure of the monoclinic polymorph of I is very close to that of the triclinic polymorph.

Apparently, the formation of the two polymorphs of I is determined by the different systems of intermolecular non-valent interactions. In the crystal of the monoclinic polymorph, the molecules are bound into the ribbons along the *c* axis by the weak intermolecular C3–H3···Cl1^i^ hydrogen bonds (Fig. 3, Table 1), whereas, in the crystal of the triclinic polymorph, the molecules form the chains along the *a* axis by the weak attractive intermolecular S···S (3.544 (3)Å) interactions (Fig. 4). Symmetry code: (i) -*x*+1, -*y*+1, -*z*.

### Experimental

A solution of SnCl_4_ (0.13 g, 0.5 mmol) in CH_2_Cl_2_ (25 ml) was added to a solution of 2,2'-dipyridyl disulfide (0.11 g, 0.5 mmol) in CH_2_Cl_2_ (25 ml) with stirring at room temperature. After 1 h, the powder of complex (C_5_H_4_NS)_2_SnCl_6_ was separated by filtration. The filtrate was concentrated in *vacuo*. The solid was re-crystallized from CH_3_CN to give I as colourless crystals. Yield is 43%. M.p. = 546-548 K. ^1^H NMR (*DMSO*-*d*_6_, 300 MHz, 302 K): δ = 8.48 (d, 2H, H6, J = 4.4), 7.81 (t, 2H, H4, J = 7.3), 7.62 (d, 2H, H3, J = 7.3), 7.28 (dd, 2H, H5, J = 7.3, J = 4.4). Anal. Calcd. for C_10_H_8_Cl_2_N_2_S_2_Sn: C, 29.29; H, 1.97; N, 6.83. Found: C, 29.21; H, 1.92; N, 6.79.

### Refinement

The crystal of I was a pseudo-merohedral twin. The twin matrix is (1 0 0 0 -1 0 -1 0 -1), and BASF is equal to 0.221 (1).

The hydrogen atoms were placed in calculated positions with C–H = 0.95Å and refined in the riding model with fixed isotropic displacement parameters *U*_iso_(H) = 1.2*U*_eq_(C).

### Crystal data

**Table:**

[SnCl_2_(C_5_H_4_NS)_2_]	*F*(000) = 792
*M**_r_* = 409.93	*D*_x_ = 2.063 Mg m^−^^3^
Monoclinic, *C*2/*c*	Mo *K*α radiation, λ = 0.71073 Å
Hall symbol: -C 2yc	Cell parameters from 6340 reflections
*a* = 6.3240 (7) Å	θ = 2.5–30.0°
*b* = 12.9391 (14) Å	µ = 2.63 mm^−^^1^
*c* = 16.4240 (18) Å	*T* = 100 K
β = 100.922 (2)°	Prism, colourless
*V* = 1319.6 (3) Å^3^	0.16 × 0.14 × 0.10 mm
*Z* = 4	

### Data collection

**Table:**

Bruker SMART 1K CCD diffractometer	1584 independent reflections
Radiation source: fine-focus sealed tube	1562 reflections with *I* > 2σ(*I*)
Graphite monochromator	*R*_int_ = 0.024
φ– and ω–scans	θ_max_ = 28.0°, θ_min_ = 1.3°
Absorption correction: multi-scan (*SADABS*; Sheldrick, 1998)	*h* = −8→8
*T*_min_ = 0.678, *T*_max_ = 0.779	*k* = −16→16
6681 measured reflections	*l* = −21→21

### Refinement

**Table:**

Refinement on *F*^2^	Primary atom site location: structure-invariant direct methods
Least-squares matrix: full	Secondary atom site location: difference Fourier map
*R*[*F*^2^ > 2σ(*F*^2^)] = 0.020	Hydrogen site location: inferred from neighbouring sites
*wR*(*F*^2^) = 0.050	H-atom parameters constrained
*S* = 1.00	*w* = 1/[σ^2^(*F*_o_^2^) + (0.014*P*)^2^ + 7.75*P*] where *P* = (*F*_o_^2^ + 2*F*_c_^2^)/3
1584 reflections	(Δ/σ)_max_ < 0.001
79 parameters	Δρ_max_ = 0.81 e Å^−^^3^
0 restraints	Δρ_min_ = −0.53 e Å^−^^3^

### Special details

**Table:**

Geometry. All s.u.'s (except the s.u. in the dihedral angle between two l.s. planes) are estimated using the full covariance matrix. The cell s.u.'s are taken into account individually in the estimation of s.u.'s in distances, angles and torsion angles; correlations between s.u.'s in cell parameters are only used when they are defined by crystal symmetry. An approximate (isotropic) treatment of cell s.u.'s is used for estimating s.u.'s involving l.s. planes.
Refinement. Refinement of *F*^2^ against ALL reflections. The weighted *R*-factor *wR* and goodness of fit *S* are based on *F*^2^, conventional *R*-factors *R* are based on *F*, with *F* set to zero for negative *F*^2^. The threshold expression of *F*^2^ > σ(*F*^2^) is used only for calculating *R*-factors(gt) *etc*. and is not relevant to the choice of reflections for refinement. *R*-factors based on *F*^2^ are statistically about twice as large as those based on *F*, and *R*-factors based on ALL data will be even larger.

### Fractional atomic coordinates and isotropic or equivalent isotropic displacement parameters (Å^2^)

**Table:**

	*x*	*y*	*z*	*U*_iso_*/*U*_eq_	
Sn1	0.5000	0.38034 (2)	0.2500	0.01554 (7)	
Cl1	0.68565 (13)	0.25722 (5)	0.18092 (4)	0.02082 (15)	
S1	0.17981 (13)	0.41891 (5)	0.14189 (4)	0.01907 (14)	
N1	0.5510 (4)	0.50833 (19)	0.16215 (15)	0.0166 (5)	
C1	0.3563 (5)	0.5095 (2)	0.11252 (18)	0.0170 (6)	
C2	0.3100 (5)	0.5780 (2)	0.04557 (18)	0.0201 (6)	
H2	0.1720	0.5786	0.0105	0.024*	
C3	0.4699 (6)	0.6444 (2)	0.0318 (2)	0.0229 (7)	
H3	0.4425	0.6915	−0.0134	0.028*	
C4	0.6725 (6)	0.6430 (2)	0.08400 (17)	0.0209 (6)	
H4	0.7836	0.6884	0.0749	0.025*	
C5	0.7064 (5)	0.5737 (2)	0.14906 (17)	0.0190 (5)	
H5	0.8425	0.5722	0.1854	0.023*	

### Atomic displacement parameters (Å^2^)

**Table:**

	*U*^11^	*U*^22^	*U*^33^	*U*^12^	*U*^13^	*U*^23^
Sn1	0.01793 (13)	0.01560 (12)	0.01225 (12)	0.0000	0.00070 (12)	0.0000
Cl1	0.0251 (4)	0.0200 (3)	0.0175 (3)	0.0044 (3)	0.0042 (3)	−0.0017 (2)
S1	0.0182 (3)	0.0197 (3)	0.0179 (3)	−0.0009 (3)	−0.0002 (3)	0.0007 (2)
N1	0.0196 (12)	0.0165 (12)	0.0131 (11)	0.0011 (9)	0.0018 (9)	−0.0001 (9)
C1	0.0209 (14)	0.0153 (13)	0.0152 (13)	0.0005 (10)	0.0041 (11)	−0.0020 (10)
C2	0.0236 (15)	0.0208 (14)	0.0150 (13)	0.0043 (12)	0.0013 (11)	−0.0019 (11)
C3	0.0334 (18)	0.0190 (14)	0.0164 (14)	0.0036 (12)	0.0046 (13)	0.0014 (11)
C4	0.0250 (15)	0.0190 (14)	0.0191 (13)	−0.0032 (13)	0.0053 (14)	−0.0012 (10)
C5	0.0213 (14)	0.0195 (13)	0.0161 (12)	−0.0007 (12)	0.0031 (12)	−0.0028 (10)

### Geometric parameters (Å, º)

**Table:**

Sn1—N1	2.259 (2)	C2—C3	1.379 (5)
Sn1—Cl1	2.3892 (8)	C2—H2	0.9500
Sn1—S1	2.4779 (8)	C3—C4	1.400 (5)
S1—C1	1.748 (3)	C3—H3	0.9500
N1—C1	1.342 (4)	C4—C5	1.380 (4)
N1—C5	1.345 (4)	C4—H4	0.9500
C1—C2	1.399 (4)	C5—H5	0.9500
			
N1^i^—Sn1—N1	85.72 (12)	N1—C1—S1	112.7 (2)
N1^i^—Sn1—Cl1	159.13 (7)	C2—C1—S1	126.3 (2)
N1—Sn1—Cl1	92.47 (7)	C3—C2—C1	118.3 (3)
Cl1^i^—Sn1—Cl1	96.36 (4)	C3—C2—H2	120.9
N1^i^—Sn1—S1	96.54 (7)	C1—C2—H2	120.9
N1—Sn1—S1	65.85 (7)	C2—C3—C4	120.4 (3)
Cl1^i^—Sn1—S1	93.80 (3)	C2—C3—H3	119.8
Cl1—Sn1—S1	101.68 (3)	C4—C3—H3	119.8
S1^i^—Sn1—S1	156.76 (4)	C5—C4—C3	118.2 (3)
C1—S1—Sn1	81.67 (10)	C5—C4—H4	120.9
C1—N1—C5	120.7 (3)	C3—C4—H4	120.9
C1—N1—Sn1	99.82 (18)	N1—C5—C4	121.4 (3)
C5—N1—Sn1	139.5 (2)	N1—C5—H5	119.3
N1—C1—C2	121.0 (3)	C4—C5—H5	119.3
			
N1^i^—Sn1—S1—C1	82.81 (12)	S1—Sn1—N1—C5	179.8 (3)
N1—Sn1—S1—C1	0.49 (12)	C5—N1—C1—C2	0.5 (4)
Cl1^i^—Sn1—S1—C1	175.74 (10)	Sn1—N1—C1—C2	−179.2 (2)
Cl1—Sn1—S1—C1	−86.96 (10)	C5—N1—C1—S1	−179.4 (2)
S1^i^—Sn1—S1—C1	43.78 (10)	Sn1—N1—C1—S1	0.9 (2)
N1^i^—Sn1—N1—C1	−99.78 (19)	Sn1—S1—C1—N1	−0.82 (19)
Cl1^i^—Sn1—N1—C1	−14.1 (3)	Sn1—S1—C1—C2	179.3 (3)
Cl1—Sn1—N1—C1	101.05 (17)	N1—C1—C2—C3	0.0 (4)
S1^i^—Sn1—N1—C1	−164.84 (16)	S1—C1—C2—C3	179.9 (2)
S1—Sn1—N1—C1	−0.64 (15)	C1—C2—C3—C4	−0.2 (5)
N1^i^—Sn1—N1—C5	80.6 (3)	C2—C3—C4—C5	−0.1 (5)
Cl1^i^—Sn1—N1—C5	166.3 (2)	C1—N1—C5—C4	−0.8 (4)
Cl1—Sn1—N1—C5	−78.5 (3)	Sn1—N1—C5—C4	178.7 (2)
S1^i^—Sn1—N1—C5	15.6 (3)	C3—C4—C5—N1	0.6 (5)

Symmetry code: (i) −*x*+1, *y*, −*z*+1/2.

### Hydrogen-bond geometry (Å, º)

**Table:**

*D*—H···*A*	*D*—H	H···*A*	*D*···*A*	*D*—H···*A*
C3—H3···Cl1^ii^	0.95	2.80	3.673 (3)	154

Symmetry code: (ii) −*x*+1, −*y*+1, −*z*.
